# Lipopolysaccharide induces the expression of an autocrine prolactin loop enhancing inflammatory response in monocytes

**DOI:** 10.1186/1476-9255-10-24

**Published:** 2013-06-03

**Authors:** Gonzalo López-Rincón, Ana L Pereira-Suárez, Susana Del Toro-Arreola, Pedro E Sánchez-Hernández, Alejandra Ochoa-Zarzosa, José Francisco Muñoz-Valle, Ciro Estrada-Chávez

**Affiliations:** 1 Unidad de Biotecnología Médica y Farmacéutica, Centro de Investigación y Asistencia en Tecnología y Diseño del Estado de Jalisco A.C. Guadalajara, Jalisco 44270, México; 2 Departamento de Microbiología e Inmunología,Facultad de Medicina Veterinaria y Zootecnia, Universidad Nacional Autónoma de México, México, DF 04510, México; 3 Departamento de Fisiología, Centro Universitario de Ciencias de la Salud, Universidad de Guadalajara, Guadalajara, Jalisco 44340, México; 4 Centro Multidisciplinario de Estudios en Biotecnología; Facultad de Medicina Veterinaria y Zootecnia, Universidad Michoacana de San Nicolás de Hidalgo, Tarímbaro, Michoacán 58893, México; 5 Departamento de Biología Molecular y Genómica, Centro Universitario de Ciencias de la Salud, Universidad de Guadalajara, Guadalajara, Jalisco 44340, México; 6 Centro de Investigación y Asistencia en Tecnología y Diseño del Estado de Jalisco A. C., Av. Normalistas 800, Colinas de la Normal, Guadalajara, Jalisco 44270, México

**Keywords:** Prolactin, Prolactin receptor, Isoforms, Monocytes, LPS

## Abstract

**Background:**

Prolactin from pituitary gland helps maintain homeostasis but it is also released in immune cells where its function is not completely understood. Pleiotropic functions of prolactin (PRL) might be mediated by different isoforms of its receptor (PRLr).

**Methods:**

The aim of this study was to investigate the relationship between the eventual synthesis of PRL and PRLr isoforms with the inflammatory response in monocytes. We used THP-1 and monocytes isolated from healthy subjects stimulated with lipopolysaccharide (LPS). Western blot, real time PCR and immunocytochemistry were performed to identify both molecules. The bioactivity of the PRL was assessed using a bioassay and ELISA to detect pro inflammatory cytokines.

**Results:**

PRLr mRNA and PRL mRNA were synthesized in THP-1 monocytes activated with LPS with peaks of 300-fold and 130-fold, respectively. The long (100 kDa) and the intermediate (50 kDa) isoforms of PRLr and big PRL (60 kDa) were time-dependent upregulated for monocytes stimulated with LPS. This expression was confirmed in monocytes from healthy subjects. The PRLr intermediate isoform and the big PRL were found soluble in the culture media and later in the nucleus in THP-1 monocytes stimulated with LPS. Big PRL released by monocytes showed bioactivity in Nb2 Cells, and both PRL and PRLr, synthesized by monocytes were related with levels of nitrites and proinflammatory citokines.

**Conclusions:**

Our results suggest the expression of a full-autocrine loop of PRL enhances the inflammatory response in activated monocytes. This response mediated by big PRL may contribute to the eradication of potential pathogens during innate immune response in monocytes but may also contribute to inflammatory disorders.

## Background

PRL is a hormone produced primarily by the anterior pituitary gland and acts on different cell types [1]. PRLr shares structures and signal transduction pathways with the type 1 cytokines and their receptors. Type I long-chain cytokines, such as IL-6, growth hormone and PRL share the JAK-STAT signal transduction pathway [2]. PRLr lacks intrinsic kinase activity and the receptor-Jak2 acts in concert to transmit signals downstream of ligand binding [3]. The main signaling networks downstream of PRLr include the Jak-STAT [4], Ras-MAPK and PI3K-AKT pathways [5]. Lymphoid cells express an autocrine loop of PRL affecting proliferation, cytokine secretion and immune activity [6-8]. However, studies with knockout mice suggest that PRL-mediated signaling is not necessary for immune response [9,10]. Nevertheless, little is known regarding the expression of extrapituitary PRL and PRLr isoforms in myeloid cells [11,12].

An acute phase response model showed differential expression of PRLr in various lymphoid and non-lymphoid organs [13]. Peritoneal macrophages (Mϕ) respond to PRL, secreting IL-1β, TNF-α and IFN-γ through the activation of the JAK2-STAT1 pathway [14]. In fibroblast treated with proinflammatory cytokines, the expression of the long isoform of the PRLr (of 100 kDa) has been associated not only with the phosphorylation of signal transducer and activator of transcription 5B (STAT5B), but also with the inhibition of the interferon regulatory factor 1 (IRF-1) and inducible nitric oxide synthase (iNOS) expression [15]. In mononuclear phagocytes, reprogramming is a regulatory process useful during inflammatory response, driven by several cytokines [16] and some hormones [17]. Although the role of PRL during inflammation has been investigated in Mϕ and fibroblast, the results might be considered controversial due to masked effects of other molecules released by differentiated inflammatory cells into the culture medium.

The PRL from pituitary gland may help to maintain homeostasis during inflammatory responses throughout the differential PRLr isoforms expression [12,15]. The expressions of PRLr isoforms have been identified in several tissues throughout the body [18], suggesting the transcriptional and posttranslational regulation of PRLr [19]. The expression of several isoforms also suggests the activation of alternative signal transduction pathways [20]. Likewise, the expression of an autocrine loop of PRL in lymphocyte [6-8] implies that PRL and its receptor (PRLr) must be synthesized and that the ligand is also secreted for the same cell; as well as, that this released PRL has a bioactivity in the synthesizing cell (e.g. proliferative responses). Although the expression of PRL in peripheral blood mononuclear cells (PBMC) has been noted [11,12], there is no complete evidence of the expression of an autocrine loop with the participation of PRL in monocytes. Moreover, the precise role and mechanism of action of PRL in mononuclear phagocytes still remains elusive. We hypothesized that the expression of an autocrine loop of PRL may play an important role during the inflammatory response in monocytes. Afterwards, the aim of this study became investigating the relationship between the eventual synthesis of PRL and PRLr isoforms with the inflammatory response elicited by LPS in monocytes.

To test this hypothesis, we used two kinds of human monocytic cells activated with LPS *in vitro*: THP1 cell line [17] and monocytes from healthy subjects, likely with different genotypes. To avoid as many masked effects in the culture media as possible, LPS doses and the bacterial strain source were experimentally determined. This was done to keep the great stimulation and the lower differentiation of the cells towards macrophage phenotype. The monocytic expression of PRLr and the local synthesis of PRL were studied at both mRNA and protein levels during response elicited by LPS, using total protein extracts, supernatants and nuclear protein extracts. The PRL bioactivity (proliferation) was assessed in lactogen-dependent Nb2 cells. We inmmunoinhibited the nitrates and citokines release by monocytes after stimulation with LPS for 48 h, using anti-PRL and anti-PRLr antibodies.

## Methods

### Reagents and antibodies

Human recombinant PRL (hrPRL) and LPS were obtained from Sigma-Aldrich (St. Louis, MO). Some antibodies were obtained from Santa Cruz Biotechnology (Santa Cruz, CA): rabbit anti-PRLr (H-300) against residues from 323-622 in the exon 10 sequence of the human PRLr, which recognize long isoform (LF), intermediate isoform (IF), delta short isoform 1 (ΔS1) and short isoform 1a (S1a); mouse IgG1anti-PRL (E-9) against residues from 96 to 200 in the exons 3, 4 and 5 of the human PRL; goat anti-mouse IgG-HRP and goat anti-rabbit IgG-HRP. Mouse IgG1 anti-PRLr (MAB1167) against human PRLr extracellular domain (R&D Systems, MN) was previously used to neutralize PRLr function [21]. A second mouse IgG1anti-PRL (6F11) that recognized an epitope restricted to the carboxyl-terminal disulfide loop conserved among prolactins from several species was used (QED Bioscience, San Diego, CA).The anti-human actin mAb was obtained from Chemicon (Temecula, CA). Green-fluorescent Alexa Fluor® 488 goat anti–mouse IgG isotype–specific, orange-Red fluorescent Alexa Fluor® 568 goat anti–rabbit IgG isotype–specific, and 4'-6-Diamidino-2-phenylindole (DAPI) were obtained from Gibco (Invitrogen Corp., Carlsbad, CA).

### Experimental procedures with different cell lines and isolated monocytes

THP-1 cells (ATCC®) were maintained in RPMI 1640 medium containing 10% (v/v) FBS and 1% (v/v) antibiotic-antimycotic at 2 × 10^5^ cell/ml as described [22]. THP-1 cells in 6-well (Nunc) or 96-well plates (Corning) cultivated for 0.5, 1, 2, 4 and 8 h were stimulated with LPS (1 μg/ml). Nb2 cells were cultivated in high-glucose D-MEM supplemented with 10% HS, 10% FBS and 10% antibiotic-antimycotic as described [23]. MCF-7 breast cancer cell line (ATCC®) was cultivated in RPMI 1640 as reported [24]. Monocytes from PBMC were isolated from heparinized (5 U/ml) blood of ten healthy male donors (29.8 ± 7.4 years old) by a standard density gradient centrifugation at 400 *g* using lymphocyte separation medium (Sigma Chemical) for 15 minutes at room temperature as described [25]. The cells at the interface were collected and washed three times in cold PBS containing 0.1% BSA. PBMC were maintained 24 h in RPMI 1640 medium containing 10% (v/v) FBS and 1% (v/v) antibiotic-antimycotic at 5 × 10^6^ cells/ml. Non-adherent cells were removed by washing in BSA-PBS and then remaining adherent cells (>95% CD14+ cells) were cultivated and stimulated 8 h with LPS (1 μg/ml). Healthy donors volunteered to participate and signed the informed consent letter before inclusion in the study. The investigation was performed according to the ethical guidelines of the 2008 Declaration of Helsinki and was approved by the ethical investigation and biosecurity committee of the University Center of Health Sciences at the University of Guadalajara. To determine the dose and source of LPS used in this study we performed dose-response assays using LPS from *Salmonella enterica* serotype Minnesota and *Escherichia coli* 0111:B4. After that, we choose the highest dose of *S. enterica* LPS for priming cells, avoiding as much as possible the differentiation of monocytes towards Mϕ phenotype.

### Nb2 cell bioassay of THP-1-treated supernatants

Supernatants were obtained by incubating non-confluent THP-1 (7 × 10^5^ cells/ml) for 1, 2, 4 and 8 h with LPS (1 μg/ml). The supernatants were concentrated 24-fold using Centricon 10 (Millipore, Billerica, MA). Nb2 cells (4 × 10^4^ cells/ml) were cultured for 60 h with serial dilutions of treated or control concentrated supernatants (5, 10, 20 and 45 μL). Nb2 cell proliferation and viability were measured with reduction of MTT as described [26]. Bioactivity was extrapolated from a standard dose-response curve with recombinant hPRL (1, 10, 100, 500 and 1,000 pg/ml). Bioactivity was inhibited with 4 μg of α-human PRL (E-9) for each dilution assayed.

### Real-time RT-PCR

Total RNA was extracted from THP-1-MO (Trizol, Invitrogen) and cDNA was synthesized (Superscript III, Invitrogen). PRLr and PRL transcripts were measured in triplicate by real-time quantitative RT-PCR using Applied Biosystem PRISM 7300 (Applied Biosystems, Foster City, CA). To amplify the conserved region of PRLrmRNA, the following forward and reverse primers were used: 5′-AGA CCA TGG ATA CTG GAG TA -3′and 5′-GGA AAG ATG CAG GTC ACC AT -3′, respectively (Primer Express; Applied Biosystems). The fluorogenic probe used for PRLr was 6FAM - TCT GCT GTC ATC TGT TTG ATT A (Applied Biosystems). To detect the PRL mRNA, exons 4-5 were amplified with the primers and the probe 6FAM corresponding to assay IDHs01062137_m1 (Applied Biosystems). The 18S ribosomal RNA (rRNA) gene (Applied Biosystems) was used as a housekeeping gene and comparative C_t_ (2^-ΔΔCt^) method for relative expression was analyzed as described [27].

### Western blot (WB) protocol and analysis

THP-1 cells or monocytes from donors were harvested, washed twice with phosphate-buffered saline (0.01 M phosphate buffered saline (NaCl 0.138 M; KCl - 0.0027 M); pH 7.4, at 25°C), and disrupted with RIPA buffer (Sigma-Aldrich, St. Louis, MO) containing 150 mM NaCl, 1.0% IGEPAL® CA-630, 0.5% sodium deoxycholate, 0.1% SDS, 50 mM Tris, pH 8.0. Next, protease (1 μM pepstatin A, 2 μM leupeptin, 0.3 μM aprotinin, 2 μM chymostatin, 2 μM antipain and 0.1 mM PMSF) and phosphatase inhibitors (0.2 mM Na3VO4 and 5 mM NaF) were added and finally clarified by centrifugation at 4°C for 20 min. Protein concentration was determined by Lowry method (BCA Protein Assay Reagent, Pierce). Total proteins 40 μg were electrophoretically separated by 10% SDS-PAGE and transferred to PVDF membrane (Bio-Rad) and blocked with 5% (wt/v) skimmed milk and 1% (wt/v) BSA. Afterwards, membranes were incubated with anti-PRLr(H-300) or anti-PRL (E-9) antibodies diluted 1:200 at 4°C overnight. HRP-conjugated anti-rabbit or anti-mouse secondary antibodies and a chemiluminescence system were used for blot development (Pierce). Intensity of bands was quantified by densitometry using Gel Logic 112 imaging system and molecular imaging software, 5.0 (Kodak, Rochester, NY). β-actin levels were determined as an internal control. WB figures are representative of three independent experiments with similar results.

### Nuclear protein extraction

THP1 cells stimulated with LPS were harvested after 48 h for the nuclear protein extraction with a kit (CelLyticTMNuCLEARTM Extraction, Sigma-Aldrich). According to the manufacturer’s instructions, cells were allowed to swell with hypotonic buffer. Once disrupted, the cytoplasmic fraction was removed and the nuclear proteins were released from the nuclei precipitated with a high salt buffer.

### Fluorescent immunocytochemistry

THP-1-MO (7.5 × 10^3^ cells/100 μL) were harvested by cytospin (Stat Spin Express 2 centrifuge, Norwood, MA), plated on Kling-on HIER slides (Biocare Medical, Concord, CA), fixed in 4% (v/v) formaldehyde at −20°C for 10 min and blocked with PBS supplemented with 10% (v/v) FBS and 1% (w/v) BSA at room temperature for 1 h. Next, cell preparations were washed and incubated with 1:50 anti-PRLr or 1:500 anti-PRL at 4°C overnight; and then incubated with 1:1,000 Alexa Fluor® 488-conjugated α-mouse or Alexa Fluor® 568-conjugated anti-rabbit for 1 h. Cell nuclei were counterstained with 1 μg/ml DAPI. Nonspecific immunolabelling was determined omitting primary antibodies. All experiments were performed in triplicate. Fluorescence microscopy was performed with an AxioImager 2 fluorescence microscope (Carl Zeiss, Göttingen, Germany). Images were captured using an AxioCamMRm camera (Carl Zeiss) and the AxioVs40 V 4.8.2.0 software (Carl Zeiss). Signal intensity was quantified using densitometry with the Imagen J 1.43 μ software from the National Institutes of Health. Images from 30 cells positive for PRL and PRLr labeling were collected and quantified; integrated density was the sum of the pixel values.

### Nitric oxide determination

The oxidation products of nitric oxide in THP1 and fresh monocytes were determined in cell culture media by the Greiss (G4410, Sigma-Aldrich) reactions as previously described [28]. Nitrite content was quantified by extrapolation from sodium nitrate standard curve in each experiment (14.4, 11.2, 8.4, 5.6, 2.8, 1.4, 0.7 μM). Normalized results after subtracting the value obtained with untreated cell cultures were considered for comparisons. The concentration of nitrite was inmunoinhibited with 4 μg of mAb anti-PRL (E-9) in monocytes cultures.

### Cytokine assays

THP1 cells were stimulated with LPS and supernatants were harvested and stored at -80°C after 48 h until analysis. Using ELISA with pre-coated plates (LEGEND MAX™, BioLegend Inc., San Diego, CA) the levels of human IL-1β (435007), human IL-6 (430507) and human TNF-α (430207) were measured according to the manufacturer’s instructions. The immunoinhibition of citokines release was performed with 10 μg of mAb anti-PRLr (MAB1167) in monocytes cultures.

### Statistical analysis

The mean values ± SD from a representative experiment are shown for samples measured in triplicate. Statistical analysis was performed with a two-way ANOVA comparing treated *versus* untreated control and measurement periods as the independent variables. The Bonferroni test was used to adjust for multiple comparisons. Data were analyzed using GraphPad Prism version 5.03 (San Diego California, USA). Significance was defined as *p*<0.05.

## Results

To choose the dose of LPS to stimulate our culture cells, avoiding the differentiation of monocytes towards Mϕ phenotype as much as possible, we performed dose-response assays using LPS from *Salmonella enterica* serotype Minnesota and *Escherichia coli* 0111:B4 (Additional file 1: Figure S1). Nitrates released indicated that THP-1 monocytes stimulated with 1 μg of LPS of *S. enterica* showed significant difference in comparison with not only lower but also higher doses (*p*<0.05) (Additional file 1: Figure S1A) after 48 h. Similar results were found using same doses of LPS of *E. coli* (*p*<0.01) (Additional file 1: Figure S1B). Cytometry analyses of THP-1 cells stimulated showed significant differences in size *versus* granularity when LPS of *E. coli* was used in comparison with LPS of *S. entrerica* (Additional file 1: Figure S1E). Whereas *E. coli* LPS induced and increased the cell size, *S. entrerica* did not. In addition, the cells maintained similar granularity compared with the untreated cells after 48 h (Additional file 1: Figure S1C). Kinetic of nitrates released showed only significant differences when LPS of *S. enterica* was used for 48 h, but not with *E. coli* (Additional file 1: Figure S1D) nor before 48 h of stimulation with either. Taking these results into account, to induce an inflammatory response we used 1 μg of LPS of *S. enterica,* maintaining undifferentiated monocytic cells. These included both the THP-1 cell line and those obtained from peripheral mononuclear blood cells of different healthy donors.

### PRLr mRNA and PRL mRNA were synthesized in THP-1 monocytes stimulated with LPS

**Figure 1:**
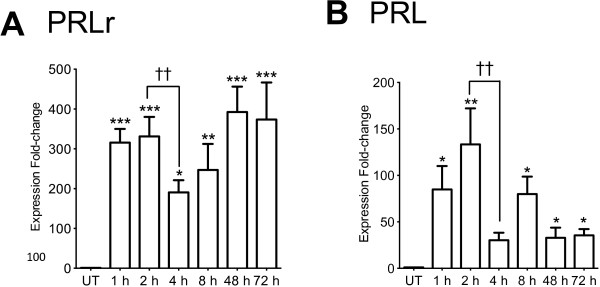
**PRLr mRNA and PRL mRNA synthesis in THP-1 monocytes activated with LPS using real-time PCR.** THP-1 monocytes stimulated with LPS 1 μg/ml were harvested at different times and three independent RT-PCR experiments were analyzed using comparative C*t* (2^-ΔΔCt^) method for: **A**, PRLr mRNA; and **B**, PRL mRNA. Data are expressed as mean ± SD, and significance *versus* untreated (UT) control is defined as *p*<0.05*, *p*<0.01**, *p*<0.001***; and between 2 *versus* 4 h *p*<0.01††.

PRLr mRNA and PRL mRNA RT-PCR assays were performed to determine if THP-1 monocytes treated with LPS (1 μg/ml) were able to synthesize. Results showed that expression of total PRLr mRNA increased over 300-fold from 1 h to 72 h (*p*<0.001) after LPS treatment, except at 4 and 8 hours when increases were 223-fold (*p*<0.05) and 247-fold (*p*<0.01), respectively (Figure 1A). The difference in the PRLr mRNA synthesized between 2 and 4 h was significant (*p*<0.01) as well. The expression of PRL mRNA increased 80-fold (*p*<0.05) and 133-fold (*p*<0.01) after 1 and 2 h of LPS stimulation, respectively. Then, PRL mRNA decreased below 30-fold (*p*<0.01) at 4 h, increased 80-fold (*p*<0.05) at 8 h, and again decreased below 30-fold after 48 h (*p*< 0.05) (Figure 1B).

### PRLr and PRL were expressed in THP-1 monocytes stimulated with LPS

**Figure 2:**
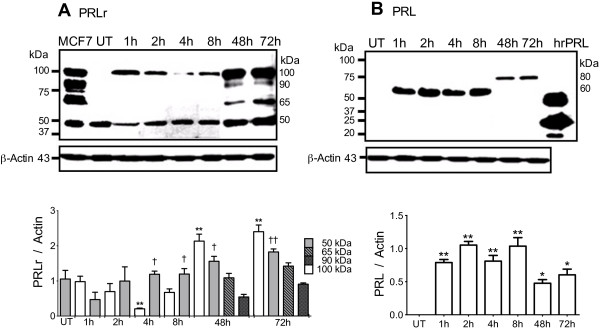
**PRLr and PRL expression in THP-1 monocytes activated with LPS using Western blot.** THP-1 monocytes stimulated with LPS 1 μg/ml were harvested at different times and two independent Western blot experiments were performed and analyzed using densitometry analysis (graph): **A**, PRLr; and **B**, PRL. As positive controls of PRLr isoforms the total protein extract from breast cancer cell line (MCF-7), showing short, intermediate and long isoforms, was included. A positive control for human recombinant PRL (hrPRL) showing 50, 23 and 16 kDa forms was included. Anendogenous control of β-Actin showing 43 kDa was revealed. Data are expressed as mean ± SD, and significance *vs.* untreated (UT) control is defined as: *p*<0.05*; *p*<0.01**; and between 1 *versus* later hours *p*<0.05†; *p*<0.01††.

Two PRLr isoforms of 100 and 50 kDa were identified in THP-1 monocytes by Western blot until 8 h of stimulation with LPS (Figure 2A). In relation to untreated cells, the expression of the 100 kDa isoform increased from 1 to 8 h, except at 4 h when the expression was significantly lower in comparison with any time after stimulation with LPS (*p*<0.01) (Figure 2A). A basal expression of the PRLr isoform of 50 kDa was observed (Figure 2A), but an increase in a time-dependent manner was found from 4 h (*p*<0.05) to 72 h (*p*<0.01) after LPS stimulation (Figure 2A). Also, a continuous increased expression of PRL of 60 kDa in THP-1 monocytes was detected from 1 to 8 h after stimulation with LPS; then, after 48 h the expression of a bigger PRL of 80 kDa was detected (Figure 2B). No basal expression of PRL was detected in untreated cells (Figure 2B).

### Isoforms of PRLr and PRL were expressed by monocytes from healthy subjects after stimulation with LPS

**Figure 3:**
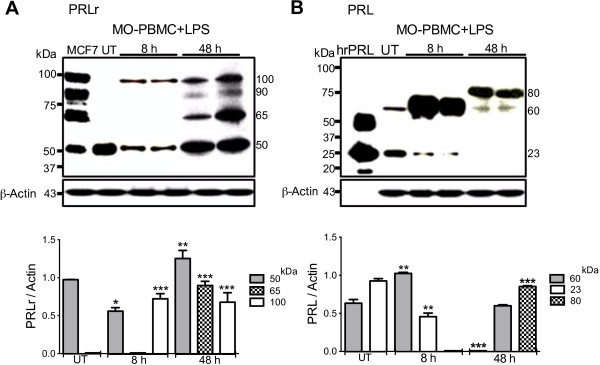
**PRLr and PRL expression in monocytes isolated from peripheral mononuclear cells (PBMC) of healthy donors.** Monocytes isolated from PBMC of different healthy male subjects were tested by Western blot analysis after activation with LPS 1 μg/ml for 8 h: **A**, PRLr; and **B**, PRL. Total extract proteins of the breast cancer cells (MCF-7) or human recombinant PRL (hrPRL) were used as positive controls. β-Actin expression was revealed as endogenous control and graphs show densitometry analyses. Experiments were performed in triplicate. Data are expressed as mean ± SD, and significance is defined as *p*<0.05*, *p*<0.01**; *p*<0.001*** *versus* control.

In untreated monocytes obtained from healthy subjects a PRLr isoform of 50 kDa (Figure 3A) and PRL of 60 kDa and 23 kDa were found (Figure 3B). Until 8 h after stimulation with LPS, the increased expression of PRLr isoforms of 50 kDa (*p*<0.05) and 100 kDa (*p*<0.001) (Figure 3A) and PRL of 60 kDa (*p*<0.01) was observed, as well as, the decrease of PRL of 23 kDa (*p*<0.01) (Figure 3B). After 48 hours of stimulation with LPS the increased expression of PRLr isoforms of 100, 90, 65 and 50 kDa (Figure 3A), and the increase of the 80 kD PRL (*p*<0.001) was revealed, as well as a decrease of PRL of 60 kDa (Figure 3B). PRL of 23 kDa was not detected after 8 h of stimulation with LPS (Figure 3B). Using a second Mab against human PRL (6F11), the expression of a PRL-like protein of 80 kDa in samples of THP-1 monocytes, as well as, monocytes derived from peripheral blood mononuclear cells of healthy donors was confirmed, using both total or nuclear protein extracts (Additional file 2: Figure S3).

### PRLr and PRL were colocalized inTHP-1 monocytes stimulated with LPS

**Figure 4:**
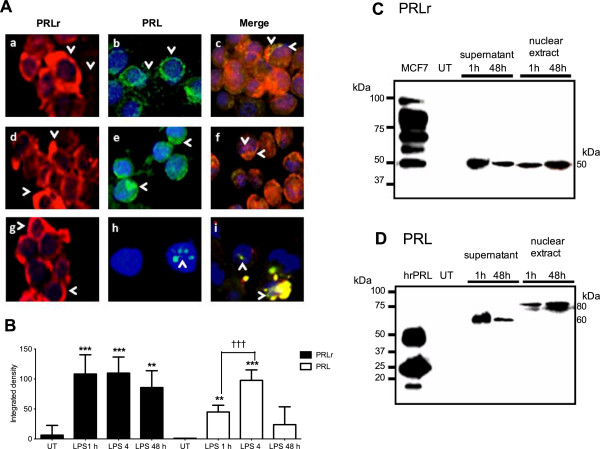
**Colocalization of PRLr and PRL in THP-1 monocytes activated with LPS. A**, fluorescent immunocytochemistry (FI) to detect PRLr and PRL was performed with THP-1 monocytes activated with LPS 1μg/ml and harvested after: 1 h, a, b and c; 4 h, d, e and f; 8 h, g, h and i. Representative results for PRLr, a, d and g; PRL, b, e and h; as well as, merged by double immunocytochemistry for PRLr and PRL are shown (1250X). The nucleus was counterstained with DAPI and an overlay was performed. **B**, cell signal intensity obtained with FI for PRLr (black) and PRL (white) was quantified using densitometry and compared between 1, 4 and 48 h after stimulation with LPS. Experiments were performed in triplicate, and significance is defined as *p*<0.001**; *p*<0.0001*** *vs.* control or between 1 and 4 h *p*<0.0001†††. **C**, Western blot analysis of PRLr; and **D**, PRL with culture supernatant and nuclear extracts after stimulation with LPS are shown. As positive controls, total MCF-7 extracts and hrPRL were used; and β-Actin was revealed as an endogenous control.

To determine the localization of PRLr and PRL, fluorescent immunocytochemistry assays were performed with THP-1 monocytes treated with LPS. A staining pattern corresponding to PRLr (in red) was localized to the surface of cells from 1 to 8 h (Figures 4Aa, Ad and Ag); a scanty label of PRLr was also observed in culture media (fixed surrounding cells) within 2 h after stimulation with LPS (Figure 4Aa). Likewise after 1 h, PRL expression in THP-1 monocytes (in green) was observed in the media and on the cell surface (Figure 4Ab). The PRL expression was observed once again after 8 h in cytoplasm (Figure 4Ae) and later in the nucleoplasm (Figure 4Ah). Using double immunolabelling colocalization of PRL and PRLr was observed in the cellular surface (in orange) (Figure 4Ac) and cytoplasm (in magenta) of THP-1 monocytes from 1 to 8 h (Figure 4Af) after stimulation with LPS; and then in the nucleoplasm (yellow) after 48 h (Figure 4Ai). Densitometry supported a significantly induced expression of PRLr (*p*<0.001) and PRL in stimulated monocytes (*p*<0.001) after 8 h in comparison with untreated cells (Figure 4B). To assess the release of soluble molecules of PRLr and PRL and its likely translocation to the nucleus, cell-free supernatants from culture media and nuclear protein extracts of THP-1 monocytes treated with LPS were analyzed. The results revealed soluble isoforms of both PRLr of 50 kDa (Figure 4C) and PRL of 60 kDa (Figure 4D) that decreased from 1 to 48 h. They also showed the translocation of PRLr isoform of 60 kDa and PRL of 80 kDa to the nucleus that increased between 1 and 48 h. In contrast neither PRLr (Figure 4C) nor PRL (Figure 4D) were found in supernatant and nuclear extract proteins of untreated cells. When primary antibodies against epitopes in the PRL and PRLr sequences were omitted, no signals were revealed by fluorescent immunocytochemistry assays using same conditions with monocytes and LPS stimulation (Additional file 3: Figure S2).

### PRL of 60 kDa released from LPS-stimulated THP-1 monocytes showed bioactivity in Nb2 Cells

**Figure 5:**
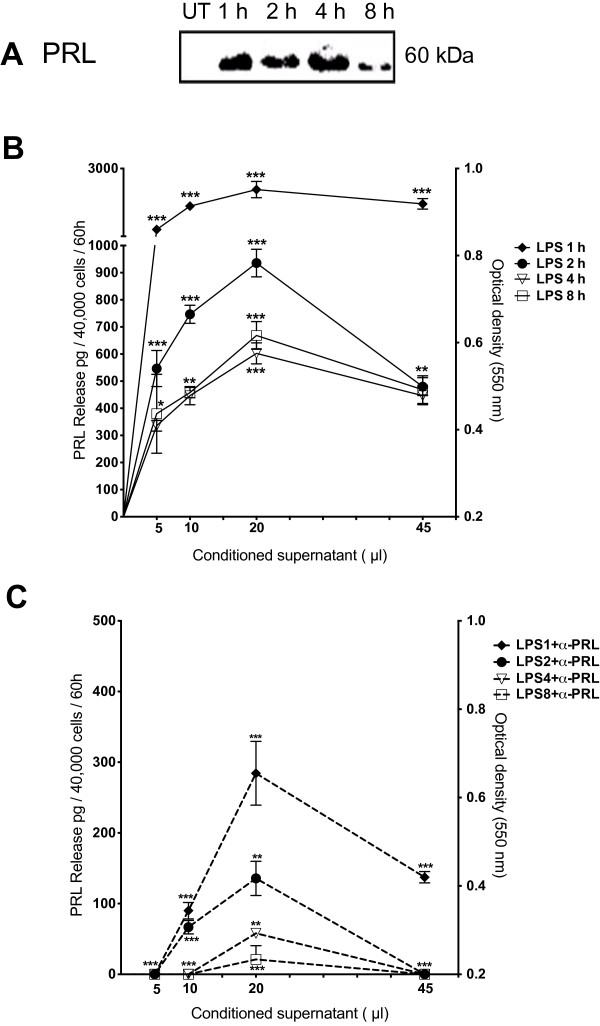
**Bioactivity of PRL of culture supernatants of THP-1 monocytes activated with LPS. A**, Western blot analysis of PRL with supernatants of THP-1 monocytes activated with LPS 1 μg/ml and harvested at different times after treatment. **B**, proliferation bioactivity in concentrated supernatants (24X) was assayed in Nb2 cell cultures after addition of different volumes: 5, 10, 20 and 45 μl; and **C**, including anti-PRL mAb E-9 (dashed line) for inmunoinhibition. Proliferation expressed as OD^550nm^ (left Y axis) or extrapolated and expressed in picograms (right Y axis) are shown. All experiments were performed in triplicate and data are expressed as mean ± SD (n = 3). Significance is defined as *p*<0.05*, *p*<0.001**, *p*<0.0001*** *versus* control.

To determine the proliferative bioactivity of PRL synthesized and released by THP-1 monocytes after stimulation with LPS, the culture supernatants were harvested from 1 to 8 h and tested in a proliferation assay with lactogen-dependent Nb2 cells. Western blot analyses of supernatants showed only PRL of 60 kDa (Figure 5A). Results of bioassay showed that bioactivity in culture supernatants decreased in a time-dependent manner from 1 to 8 h (Figure 5B); and differences between early supernatants of 1 h compared with later results of 2, 4 and 8 h were significant (p<0.001) with every volume assayed; and with 20 μl equivalents to 2 ng/ml (*p*<0.001) (Figure 5B). Using a volume of 20 μl and supernatants of 2, 4 and 8 h, the highest values of bioactivity were observed, equivalent to 900 pg/ml of hrPRL (Figure 5B); and using higher volumes of 45 μl, lower values were observed (500 pg/ml; *p*<0.01). Significant immunoinhibition of PRL bioactivity was demonstrated after the addition of mAb anti-PRL (E-9) to each of the supernatants assayed (*p*<0.001) (Figure 5C).

### PRL and PRLr synthesized by monocytes were related with nitrites and proinflammatory cytokines

**Figure 6:**
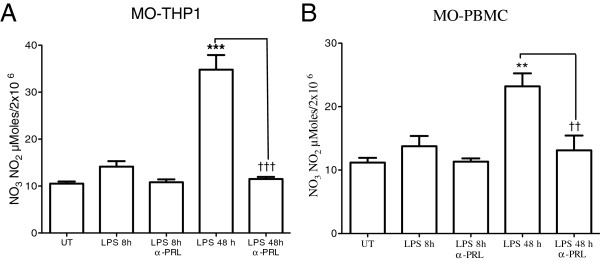
**Inhibition of nitrite response of monocytes activated with LPS using a mAb anti-PRL.** NO^3^ and NO^2^ were assayed in culture supernatants of monocytes at 8 and 48 h after stimulation with of LPS 1 μg/ml and, as well as with mAb anti-PRL (E-9): **A**, THP-1; and **B**, fresh monocytes from different subjects. Experiments were performed in triplicate. Data are expressed as mean ± SD, and significance *versus* UT control is considered as *p*<0.001***; *p*<0.01**; or between cell cultures treated with LPS alone *versus* LPS and mAb anti-PRL together, as *p*<0.01††; *p*<0.001†††.

**Figure 7:**
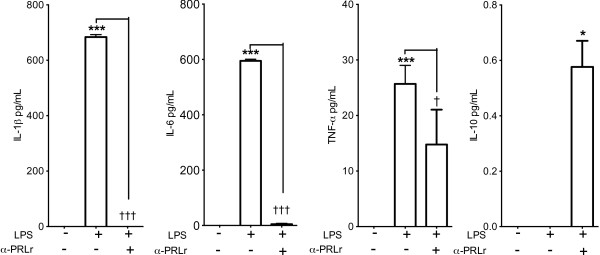
**Inhibition of proinflammatory cytokine response of THP-1 monocytes activated with LPS using a pAb anti-PRLr.** IL-1β, IL-6, TNF-a and IL-10 were assayed in culture supernatants of THP-1 monocytes activated with LPS alone or including pAb anti-PRLr (MAB1167) after 48 h. Differences among untreated cells *vs.* LPS treated are considered significant as *p*<0.05*; *p*<0.001***; or between cell cultures treated with LPS alone *versus* LPS and pAb anti-PRLr as *p*<0.05†; *p*<0.001†††.

Basal levels of nitrates were found in the culture supernatants of stimulated monocytes with LPS. They were harvested at 8 h (10 μMoles) for both THP-1 and fresh isolates from healthy subjects (Figure 6). After 48 h of stimulation with LPS nitrite, the concentration increased 3 times in THP-1 (over 30 μMoles) (*p*<0.001) and 2 times in fresh monocytes (*p*<0.01). The concentration of nitrite was inmunoinhibited in the presence of mAb–PRL (E-9) for THP-1 monocytes (*p*<0.001) and fresh monocytes (*p*<0.01) (Figure 6). In THP-1 monocytes, an increase in the release of cytokines IL-1β (>600 pg/ml) (*p*<0.001), IL-6 (600 pg/ml) (*p*<0.001) and TNF-α (<25 pg/ml) (*p*<0.001) was also seen 48 h after stimulation with LPS, and no IL-10 was detected (Figure 7). The immunoinhibition with a pAb anti-PRLr (MAB1167) completely struck the levels of IL-1β (*p*<0.001) and IL-6 (*p*<0.001), reducing the TNF-α concentration of 0.6 times (~15 pg/ml) (*p*<0.05), and inducing release of IL-10 (~ 0.5 pg/ml) (*p*<0.05) (Figure 7).

## Discussion

Ubiquitous expression of PRLr and PRL mRNA in T lymphocytes has suggested possible autocrine or paracrine PRL immune effects [6]. However, results obtained with the knockout approach pointed out an insignificant role of the PRLr and PRL in the immune response maintenance [9,10]. Therefore, these molecules might be involved in the biology of other immune cells distinct from lymphoid, e.g. myeloid cells [29]. Indeed, some reports indicate that PRL from the pituitary gland induces production of nitric oxide and TNF-α in murine peritoneal Mϕ, a process involving protein tyrosine kinases, MAP kinases and Ca++ channeling [30]. On the other hand, the inhibition of inducible nitric oxide synthase expression by pituitary PRL has previously correlated with JAK-STAT-5b activation and the suppression of IRF-1 in lung fibroblasts [15]. Using an acute inflammation model induced with LPS in mice and characterized by proinflammatory cytokine synthesis, it has been shown that PRLr mRNA is differentially expressed [13]. Nevertheless, the same cytokines induce the expression of PRLr isoforms that may allow PRL to inhibit the nitrosative stress in pulmonary fibroblasts [15].

Therefore, we hypothesized that the expression of an autocrine loop of PRL might play an important role during the inflammatory response in monocytes. To study the inflammatory response with LPS, the human monocytic leukemia-derived THP-1 cell differentiated with phorbolmyristate has been useful [22,31]. LPS is instantaneously recognized by TLR4 expressed by monocytes [22,31,32]. TLR4 is associated with MD-2 on the cell surface and this is required for induction of inflammatory cytokines. Additionally, LPS-binding protein (LBP) and CD14 are involved in the responses to LPS. CD14 binds LBP and delivers LPS-LBP to the TLR4-MD-2 complex. TLR4 is known to activate two signaling pathways: the myeloid differentiation primary response gene 88-dependent pathways and the TIR-containing adapter inducing IFNβ-dependent pathway. Signaling pathways via TLR4 mediated by these adapter molecules conclude in the activation of NF-*k*B, and/or mitogen-activated protein kinases, and/or the transcription factor IFN regulatory factor 3. Activation of these molecules regulates the expression of diverse inflammatory genes as type I IFN [33]. LPS is a specific ligand for TLR4, but cytokines and hormones may cooperate to enhance eradication of pathogens from the circulation system and tissue sites [34]. In this work, in order to avoid masked effects of other molecules released by differentiated Mϕ into the culture medium, we used undifferentiated monocytes including the cell culture line THP-1 and fresh monocytes isolated from subjects likely with different genotypes.

We used primers to amplify the conserved region of PRLr mRNA from all isoforms that retain 175 bp of the exons 7, 8 and 9 (GenBank ID: NM_001204315.1), including: LF, intermediate isoform (IF), and short isoforms, ΔSF1, SF1a, SF1b, Δ4-SF1b, Δ4/6-SF1a, SF1c; and to detect the PRL mRNA, exons 4-5 were amplified. For inmunoassays to detect the PRL and PRLr proteins, we used the mAb E-9 against an epitope in the PRL sequences, as well as the polyclonal antibody (pAb) H-300 against an epitope in PRLr sequence. The pAb H-300 was recently deemed specific and useful to perform immunehystochemical assays in human tissue samples [35]. In the case of anti-PRL, we chose the mAb E-9 against a conserved sequence of PRL, previously characterized by others [36-40].

The synthesis and expression of PRLr isoforms of 50 and 100 kDA and the isoform PRL of 60 kDA were demonstrated in THP-1 monocytes activated with LPS in this work, using transcriptional and translational approaches as previously described by others [6,27,35]. The LF of 100 kDa was inversely related with the IF of 50 kDa that was expressed at basal levels. This differential expression suggests a transcriptional regulation of the LF and IF as described in breast carcinoma cells [41]. However, these isoforms might be also posttranslationally regulated [42] and proteolysis of the LF can produce shorter isoforms [43]. The IF has been proposed to mediate diverse PRL functions in other cells [44]. In addition to the structural diversity of PRLr, different concentration-dependent functions may exist.

Our results were also supported using monocytes from healthy subjects. Other isoforms of PRLr of 90 and 65 kDa and PRL of 23 kDa were also detected after stimulation with LPS. Long (90 kDa) and short (42 kDa) PRLr isoforms resulting from differential splicing [45] and an intermediate isoform (65 kDa) from an in-frame truncation have been previously reported in other cell lineages, including human mammary tumor [46]. Previous findings suggest that PRL from the pituitary gland might help to maintain homeostasis during inflammatory responses through the differential expression of PRLr isoforms [12,15,47]. The dominant negative character of short PRLr isoforms (40 kDa) has been described in human cells [19], whereas the LF (100 kDa) and the IF (50 kDa) might trigger different signaling pathways [42]. Indeed, human prolactin receptor (PRLr) transcripts and their protein products exhibit heterogenic structures and functions [42], and at least ten isoforms of human PRLr have been identified [18,48]. Short form homodimers and long and short form heterodimers were found constitutively present in humans. These mediate the activation of JAK2, but are unable to signal through JAK2/STAT5 [20]. In addition to modulating signaling by PRLr heterodimerization, short forms might activate distinct signaling pathways [49].

The molecular heterogeneity of PRL has been described [50] and in this work we demonstrated that the monocytes activated increased synthesis and expression of a big PRL of 60 kDa. This big PRL has been previously characterized in serum, plasma, PBMC and lymphocytes from human subjects [6,51,52]. The big PRL is a dimer of covalently-linked glycosylated subunits (25 kDa) [51] with reduced biological activity [53] and might be involved in mechanisms of secretion, storage [54] and proteolysis [51]. In this work, a high proliferative bioactivity of the PRL of 60 kDa was demonstrated using lower volumes in a bioassay, suggesting the saturation of receptors available in the bioassay system. These results agree with what is known about Nb2 cells in which PRLr is abundant and only partial occupancy on the surface is required to reach maximal proliferative bioactivity [55].

In addition, we demonstrate that PRL and PRLr synthesized by monocytes activated with LPS were related with the production of nitric oxide and proinflammatory citokines (IL-1β, IL-6 and TNF-α), since the secretion of these molecules was inhibited using primary antibodies that recognized both PRL and PRLr. The PRL of 23 kDa was not found in monocytes activated with LPS after 8 h, but it was revealed in fresh untreated monocytes from different subjects. Therefore, this 23 kDa PRL was likely released from pituitary gland and transient bound to the PRLr in the monocyte. In a previous report, the activation of monocytes with both LPS and high concentrations of PRL mimics physiological hyperprolactinemic states, such as during pregnancy, promoting proinflammatory responses via NF-*k*B and IRF-1, as well as IL-10 release [56]. In this work, IL-10 was neither released by untreated nor LPS-activated monocytes, but, in contrast, the binding of PRLr with a pAb anti-PRLr elicited IL-10 in activated monocytes after 48 h. Induced production of IL-10 in LPS-activated monocytes/macrophages seems to be regulated by a direct downstream effector kinase (serine/threonine) of PI3K [57-60]. The PI3K-AKT signaling pathway plays a role in regulating cellular growth, differentiation, adhesion, and inflammatory responses. Taking the background into account, AKT activation elicited by bound PRLr (65 kDa) was probably responsible for IL-10 production and subsequent IL1-β, IL-6, TNF-α and nitric oxide drop. Recently, the activation of the human PRL extrapituitary promoter in monocytes activated with LPS was noticed as being greatly regulated and involved with the resolutive phase of inflammation [61]. However, the big PRL has been formerly correlated with the course of several inflammatory disorders [29,62]. Our results suggest that monocytes might contribute as a source of PRL found in sera patients that have chronic systemic inflammation.

Molecular colocalization performed by fluorescent immunocytochemistry assays suggests the interaction of PRLr with big PRL synthetized by monocytes after activation with LPS. The interaction might not only take place in the surface of the cells, but also at an early point in the milieu and later in the cytoplasm of activated monocytes. In addition, we demonstrate a time-dependent nuclear translocation of the PRL that had a molecular weight of 80 kDa instead of 60 kDA, as well as a PRLr isoform of 50 kDa. It is possible that the PRL of 80 kDa corresponds to a protein complex of big PRL of 60 kDA covalently linked with a PRL-interacting protein [6,51-53]. Therefore as previously proposed in cancer cells [63,64], ligand-induced complex imported to the nuclei might mediate some genetic effects of PRL in activated monocytes.

## Conclusions

Our results suggest that the expression of a full-autocrine loop of PRL enhances the inflammatory response in activated monocytes. This response mediated by big PRL might contribute to the eradication of potential pathogens during innate immune response in monocytes. In addition, this autocrine loop might prevent the resolution of systemic inflammation during inflammatory disorders in humans. Further analyses are needed to characterize the molecular mechanism regulating this autocrine PRL loop in monocytes.

## Competing interests

The authors declare that there is no conflict of interest that could be perceived as prejudicing the impartiality of the reported research.

## Authors’ contributions

GLR: Conception and design, acquisition, analysis and interpretation of data by Real time PCR, Western blot and bioassay; ALPS: Conception and design of Western blot assays, acquisition, analysis and interpretation of data; SDTA and PESH: Conception and design of experiments with cell culture line 1 and analysis and interpretation of data; AOZ: Conception and design of bioassay, and involved in drafting the manuscript; JFMV: Analysis and interpretation of data and involved in drafting the manuscript; CEC: Conception and design, acquisition, analysis, interpretation of data and drafting the manuscript. All authors read and approved the final manuscript.

## Supplementary Material

Additional file 1: Figure S1 Nitrite response assays using different doses of LPS to activate THP-1 monocytes. NO^3^ and NO^2^ were assayed in supernatants of THP-1 monocytes stimulated with different doses of LPS after 48 h. **A**, results with LPS of *Salmonella enterica* serotype Minnesota; and **B**, of *Escherichia coli* 0111: B4 strain. **C**, flow cytometry forward (FSC) and side scatters (SSC) of THP-1 monocytes untreated (UT), stimulated with LPS of *S. enterica* (1 μg/ml) and LPS of *E. coli* (1 μg/ml). NO^3^ and NO^2^ were assayed in supernatants of THP-1 monocytes stimulated with LPS 1 μg/ml and harvested at different times with: **D**, LPS of *S. enterica* and E, LPS of *E. coli*. Experiments were performed in triplicate, data expressed are as mean ± SD and significance is defined *versus *UT control as *p*<0.05*; *p*<0.01**. Click here for file

Additional file 2: Figure S3 Expression of big PRL of 80 kDa in peripheral blood mononuclear cell-derived monocytes (MO-PBMC) and THP-1 monocytes (MO-THP1). Monocytes were stimulated with LPS of *Salmonella enterica* serotype Minnesota and after 48 h, **A**) total protein extracts, or **B**) nuclear protein extracts were assayed by Western blot, using a second Mab IgG1anti-PRL (6F11). UT, untreated cells; hrPRL, human recombinant PRL; β-actina, as internal control. Click here for file

Additional file 3: Figure S2 Colocalization of PRLr and PRL in THP-1 monocytes activated with LPS. Representative fluorescent immunocytochemistry (FI) using THP-1 monocytes untreated (UT) or activated with LPS 1μg/ml (500X) and harvested after: **A**, 1 h; **B**, 4 h; and **C**, 48 h; to detect PRLr (a), PRL (b), as well as double immunocytochemistry for both (c). Negative control showing nonspecific immunolabelling was determined by omitting primary antibodies in each experiment performed in triplicate. Click here for file
